# Temporal and Spatial Structure of Collective Pass-Chaining Action Performed by Japanese Top-Level Field Hockey Players

**DOI:** 10.3389/fspor.2022.867743

**Published:** 2022-04-12

**Authors:** Takayasu Mizawa, Motoki Okumura, Akifumi Kijima

**Affiliations:** ^1^Faculty of Sport Sciences, Yamanashi Gakuin University, Kofu, Japan; ^2^Faculty of Education, Tokyo Gakugei University, Koganei, Japan; ^3^Faculty of Education, University of Yamanashi, Kofu, Japan

**Keywords:** ball game, expertise, passing action, defensive pressure, dynamical systems, temporal–spatial constraint

## Abstract

In real hockey or soccer games, scoring opportunities usually occur quite rarely, and thus, for most of the duration of a game, the ball is drifting between the two goals. This pass-chaining situation can be regarded as the stable state of the offense–defense interaction. In the current study, temporal and spatial structure of this dynamical state was unveiled *via* quantification of the “defensive pressure distribution” on the pass trajectory, which was modeled as a non-linear function of the distance between the defender(s) and a given position on the pitch. Two groups, i.e., a top-level group and a less-skilled group, of Japanese collegiate hockey players were asked to play in 3-on-3 small-sided games between players of the same skill levels. When both the top-level and less-skilled players succeeded in passing the ball, there were no skill-level differences observed in the defensive pressure distribution on the pass trajectory. In these cases, the defenders put a certain level of pressure on the middle of the pass trajectory when the passer received a previously released pass, and later, when he released the ball to a teammate, the defenders approached the position at which the passer released the ball to intensively press on the passer. However, in the cases wherein they failed to thread the ball, clear differences were observed between the groups in terms of the defensive pressure distribution on the pass trajectory. In particular, for the less-skilled group, extremely intensive defensive pressure was put on the overall regions of the pass trajectory heavily concentrated on the timing at which the passer released the pass. This unique pressure distribution emerged for the less-skilled group because of their long ball-keeping duration (longer than 1 s and also longer than that for the top-level group), i.e., from the moment the passer received the ball, to the moment he released it to the next attacker. Thus, for top-level hockey players, a short time constant (less than 1 s) for the passing action will enable the passers to avoid extremely intensive defensive pressure, and enable the emergence of a dynamically stable attack–defense deadlock state through continuous chaining of the pass.

## Introduction

### The Deadlock State in Collective Behavior in Team Sports

In a 1-on-1 combat game, the fighters' step-in and step-away movements are coupled in an anti-phase manner (Kijima et al., 2012). In national-level kendo games, the players' stepping movements are coupled in the in-phase (i.e., the player steps in/away when the opponent steps in/away) when the interpersonal distance increases by more than a certain level (Okumura et al., 2012), whereas the coupling pattern transfers to the anti-phase (i.e., the player steps in/away when the opponent steps away/in) when the distance decreases to a certain level (Yamamoto et al., 2013; Okumura et al., 2017).

Such an orderly pattern also governs collective behavior in team sports. For example, in soccer games, based on such orderly fluctuation of the attack–defense collective behavior, an orderly pattern continuously emerges in the ball behavior, persisting for no more than several tens of seconds (Kijima et al., 2014). These findings involve suggestions about the temporal order of game momentum, which are often intriguing and interesting to team sports researchers (Vallerand et al., 1988; Araújo and Bourbousson, 2016; Passos et al., 2016). Because the game momentum fluctuates in cycles of tens of seconds, as Kijima et al. (2014) suggests, it will be reasonable to hypothesize that the temporal order of the pass chaining/breaking process would be shorter than several seconds.

In addition, the exercise intensities of the players are relatively low, i.e., in the levels of walking or jogging, and rarely increase drastically, whereas the fluctuation of the pass-chaining state is moderate (Spencer et al., 2004; Sunderland et al., 2006; Macutkiewicz and Sunderland, 2011). The robustness of the teamwork to continuously stabilize such momentum differs between teams with different skill levels. Skilled teams exert high “cooperative force,” i.e., one of the three social forces, which include “spatial force” and “avoiding force,” to continuously bond the players' movement coordination (Helbing and Molnar, 1995; Yokoyama et al., 2018, 2020). The equilibrium state of the attacker–defender force is relatively stable when the attackers chain the pass while the attacking/defending team performs alterations in a phasic manner. On the other hand, an attacker breaks the state by carrying or threading the ball to completely penetrate the other side. Suppose an attacker rushes to penetrate the aperture between two defensive players. If the aperture spreads more than 4 m (Passos et al., 2008), and at the same time, the attacker's rushing speed exceeds 1 m/s, the state critically fluctuates and the probability of the attackers penetrating the defenses significantly increases (Correia et al., 2012). Similar orderly patterns of the attacking/defending team performing alterations were frequently reported in the research on the dynamical structure of the team game structure (Davids et al., 2006; Clemente et al., 2013; Morgan et al., 2013). The attacking side makes efforts to continuously create such non-equivalence to penetrate the defending side, whereas the defenders act to prevent such efforts from succeeding. Therefore, as in soccer games, the attack–defense equivalence continuously fluctuates, because of the coordination of at least three attackers, which is necessary to chain the pass against the defenders' preventative actions (Yamamoto and Yokoyama, 2011). If the team intercepts the ball beyond the enemy line, this attack–defense deadlock state tends to fluctuate significantly, and the probability of a shot or even of a score tends to increase (Sunderland et al., 2006). Most research studies on ball games have focused on teamwork that leads to a shot or a chance to score (Mosquera et al., 2007; Ng et al., 2018). On the other hand, the temporal and spatial structure of the dynamical bifurcation of the players' collective pass-chaining action that stabilize or break the attack–defense pressure equilibrium has not yet been investigated.

### Attacker–Defender Distance Functional to Stabilize/Break Pass Chain

In an expert combat game, the order of the deadlock status tends to be dynamically stable. In this status, each of the two players repeats the execution of attacking/avoiding actions at every moment; therefore, the status continuously fluctuates until the game is over (Yamamoto et al., 2013; Okumura et al., 2017). Similar to expert 1-on-1 combat game players, expert soccer teams frequently thread the pass, and thus, the ball is less likely to be intercepted by the opponent compared to in games with less-skilled teams (Dellal et al., 2011; Vinson and Peters, 2016). At first sight, a system structured by the collective behavior of the two teams in the game seems to freeze with any fluctuation; however, the frequency of within-team pass exchanges is rather higher in expert games than in games performed by less-skilled players. Therefore, the threading/intercepting of passes within/between the team is functionally the same as a step-in action in 1-on-1 combat sports, and in both cases, the players or teams continuously exchange the attacking/defending roles. Consequently, this deadlock state is maintained by the repetitive collective action to invade/pullback the space between the players or teams. In past studies, the temporal and spatial structure of such a dynamical state has not yet been investigated, although the theoretical framework of the system has been frequently proposed (Araújo and Bourbousson, 2016; Passos et al., 2016).

The functional distance that enables the emergence of attack–pullback interpersonal action coupling is determined based on the lengths of the limbs or tools used to hit the opponent's body. In combat sports such as kendo or karate (or fencing or boxing), there is a distance for each player within which his/her opponent is reachable. The distance for the emergence of the deadlock state is close to this functional distance required to reach the opponent. Similar to kendo players using a shinai, hockey players use a 1-m stick to control the ball at their feet and to attack the ball at their opponents' feet. Such a perspective as an action possibility (i.e., affordance) would fit the theoretical perspective of the ball game behavior that have been proposed based on the ecological dynamics (Araújo et al., 2016).

We quantified this functional distance of defenders to reach their opponents' ball using a concept referred to as “pressure distribution,” which is a function of the effect of each defensive player on each position on the pitch. The non-linear function of the pressure represents the “reachableness” of each defense player based on the distance from each defender [see Additional Requirements for a brief explanation and Kijima et al. (2014) for more details]. Thus, we quantified the functional distance for the defenders to be able to attack the opponents' ball and elucidated the order that regulates the deadlock state. Furthermore, we focused on top-level players, including expert players at the national level, and slightly less-skilled players, and inferred two characteristics: the dynamical structure of the pass-chaining action common to these two groups, and the essentially different structure in the process of breaking the pass-chaining action of the attacking side. The dynamics of the ball game intrinsically include a large number of elements; therefore, the spatial–temporal patterns of these dynamics are quite difficult to analyze.

We attempted to address this difficulty by introducing the collective variable of pressure distribution, which simultaneously represents affordance for the attackers (defenders) to thread (intercept) the pass in order to unveil quantitative parameters to chain or break the pass penetrating to the defensive area. The findings of this study would enhance existing knowledge regarding the mechanism of bifurcation to break a shot or a scoring opportunity.

## Materials and Methods

### Participants

Twenty-four field hockey players belonging to the Yamanashi Gakuin field hockey club were recruited for the experiment. The team won the Japanese collegiate hockey league championship seven times from 2010 to 2021. Twelve players playing as the starting members were categorized into the expert (Ex) group. On the other hand, players with no experience playing as the starter but with experience playing in official games in the league were categorized into the intermediate (Im) group. Six of the Ex group players have had experience of being invited to the National Japanese hockey team, which ranked 15th in the International Hockey Federation 2020. Two coaches estimated the ball control skills of the Ex and Im group players, specifically in switching from receiving action to passing action, based on a five-point Likert rating scale (1 = extremely poor, 2 = poor, 3 = mediocre, 4 = superior, 5 = extremely superior). The differences between the groups with regard to these four profiles were tested for significance using the Mann–Whitney U test; however, no significant differences were observed between the groups, including in terms of ball control skill. Thus, the ball control skills in simple playing-catch situations were similar between the two groups. Details were shown in Table 1.

**Table 1:** Profiles of the participants.

**Group**	**Age (yrs)**	**Height (cm)**	**Weight (kg)**	**Skill**
Ex	20.50 ± 0.96	170.67 ± 5.72	64.50 ± 5.88	3.38 ± 0.51
Im	19.92 ± 1.19	170.58 ± 5.25	64.92 ± 5.33	2.96 ± 0.48

*Mean and standard deviation of each group. Skill: Score of ball control skill. The score for each player was calculated as the average of scores from two coaches at Yamanashi-Gakuin University. No significant group differences were observed for any of the variables*.

### Experimental Task and Procedures

Four teams consisting of three members were set up for each of the two groups, equalizing the numbers of player positions. The goal keepers were not recruited and this position was excluded from the game. Each of the four teams in each group were asked to play in a round-robin competition with the three other teams of the same skill level. More specifically, each of the four teams in each skill-level group was asked to play a 3-on-3 small-sided game twice with each of the three other teams; therefore, each team played the game six times. Each game was played for two minutes. The pitch was designed to have dimensions of 30 m × 18 m, which is approximately one-third of the official hockey pitch size. This sizing enabled the imitation of the density around the goal as in an official hockey game. Each team's goal was set at the center of their end line. The players were allowed to shoot only from the opponents' side beyond the center line. The ball was restarted from the same position when the ball crossed the line on the defender's side; however, when the ball crossed the line on the attacker's side, the ball was restarted from the crossing point of the center line and sideline. The players were allowed to pass or shoot by a grounded ball but were not allowed to float the ball to avoid injury. When a player floated the ball over 30 cm in height, the ball possession team was changed. This penalty was applied only once in all 24 games performed by each of the two skill-level groups. The pitch was a water-based artificial turf. All games were performed under clear weather conditions. All methods were performed in accordance with declaration of Helsinki and the game was conducted as part of the training program of the 1st and 2nd teams of Yamanashi Gakuin field hockey club. Informed consent was obtained from all participants using the procedure approved by the Research Ethics Committee of the Faculty of Education, University of Yamanashi.

### Measurement of 2D Positions of Ball and Players

**Figure 1:**
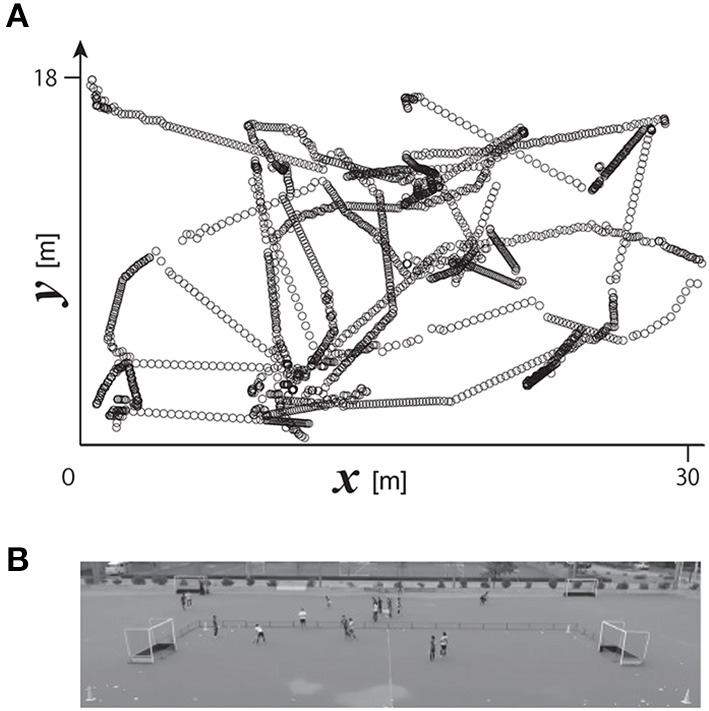
**(A)** Typical example of ball movement on a 2D plane. **(B)** Birds' eye view of the pitch recorded using a video camera.

A digital video camera (HDR-CX720, Sony; 30 Hz) was set on the spectators' stand in Yamanashi Gakuin Hockey Stadium to cover the overall area of the pitch of the small-sided game (see Figure 1A). The position of the camera was fixed while the game was progressing. The reference points for calculating the camera parameters were set on positions every five meters on each end line on both sides (parallel to the *y* axis), and six meters on each sideline on both sides (parallel to the *x* axis). Ten crossing points on lines connecting pairs of diagonal points on both the sidelines and end lines were added; therefore, the number of reference points for calculating the camera parameters was 28 in total. The 2D positions of the ball and of the six players' feet on the pitch were calculated using the 2D direct linear transformation method. Each player's 2D position on the ground was defined as the position of his grounded foot when only one of his feet was grounded; on the other hand, when both of his feet were simultaneously on the ground, the player's position was defined based on the mean vector of both grounded feet. A series of calculations was performed using motion capture software (Frame Dias V, DKH Inc., Japan). A typical example of the 2D trajectories of the ball is shown in Figure 1B.

### Dependent Measures and Statistical Procedures

#### In-play Time

The in-play time was defined as the duration during which the players moved the ball on the pitch, except for the times during which the ball went out of bounds or when the game was stopped by an incident such as a rule violation. The time was measured at a temporal frequency of 30 Hz using an event coding system (Sports Code GAMEBREAKER+, Hudl Corp. Inc.). The significance of the group difference was tested using the Mann–Whitney U test. The effect size was estimated using *r*, and when it was equal to or larger than 0.4, we assumed that the effect was sufficiently large (Funder and Ozer, 2019).

#### Technical Action

The frequency at which an attacker succeeded or failed to thread the pass to a player of the same team was counted using the event coding system. The frequency of the turnover was also counted *via* summation of the frequencies of intercepting and stealing the ball by the defense team, although the number of turnovers due to the ball going out of bounds was excluded from this measure. The successful-pass frequency indicates the frequency of the ball traveling within a team, representing the frequency of the smaller within-team fluctuation of the states. On the other hand, the failed-pass frequency or the frequency of turnovers indicates the frequency of the ball traveling between the two teams, representing the larger between-team fluctuation of the states. The significances of the group differences were tested using the Mann–Whitney U test. Similarly to the analysis of in-play time, when the effect size *r* equal to or larger than 0.4, we assumed that it indicate a sufficiently large effect. Details were shown in Table 2.

**Table 2:** Number of technical actions.

**Group**	**Time of in-play (s)**	**N.Succ.pass** ^*^	**N.Fail.pass** ^*^	**Shots** ^*^	**Turnovers**
Ex	56.96 ± 9.29	16.25 ± 2.20	3.75 ± 1.36	3.75 ± 1.53	3.92 ± 1.80
Im	58.42 ± 7.99	12.08 ± 2.02	5.50 ± 1.71	2.33 ± 0.85	4.17 ± 1.28

*Mean and standard deviation for each group. N.Succ.pass: Number of successful passes. N.Fail.pass: Number of failed passes*.

* *Significant difference (p < .05) between groups*.

#### Ball-Keeping Duration

If the time during which a passer holds the ball around his/her feet is extended, it becomes easier for defenders to approach the passer, and the trajectory of the pass will be restricted. This is, especially true in a small-sided game played in a cramped pitch, where pass efficiency will be seriously deteriorated when the time during which the passer keeps the ball is increased. In this study, we defined this duration as the ball-keeping duration, which starts at the moment at which the player contacted their stick with the ball to receive it, and ends at the moment at which the ball was released from the stick to shoot a pass. The ball-keeping duration was measured at a frequency of 30 Hz based on the number of frames of the video clip. The data of the duration measured for all passes, including both successful and failed passes, were pooled for each of the 24 games. We then calculated the histogram, which visualizes how the frequency of the pass differed throughout the ball-keeping duration. The duration (bins) of the histogram was divided into seven regions, i.e., 0–1, 1–2,… to 6–7 s, because the longest ball-keeping duration for a successful pass was 7 s. A two-way mixed design ANOVA involving the groups (2: Ex, Im) and ball-keeping regions (7: 0–1, 1–2,… to 6–7 s) was conducted to test the significance of the effects of the groups and regions. When any violations to sphericity were found, we collected *p*-values using the Greenhouse-Geisser procedure. The Bonferroni method was used for multiple comparison. The explanation of the main effect of each group and duration region was excluded, when the effect of the interaction was significant, because the main focus of the analysis was the interaction between the two factors, particularly the between-group difference in the distributions of the ball-keeping durations. The the effect size was estimated using $\eta_{p}^{2}$, and when $\eta_{p}^{2}$ was equal to or larger than 0.14 and 0.06, we assumed that it indicated a sufficiently large and medium effect, respectively (Field, 2013).

#### Quantification of Defensive Pressure Distribution on Pass Trajectory

**Figure 2:**
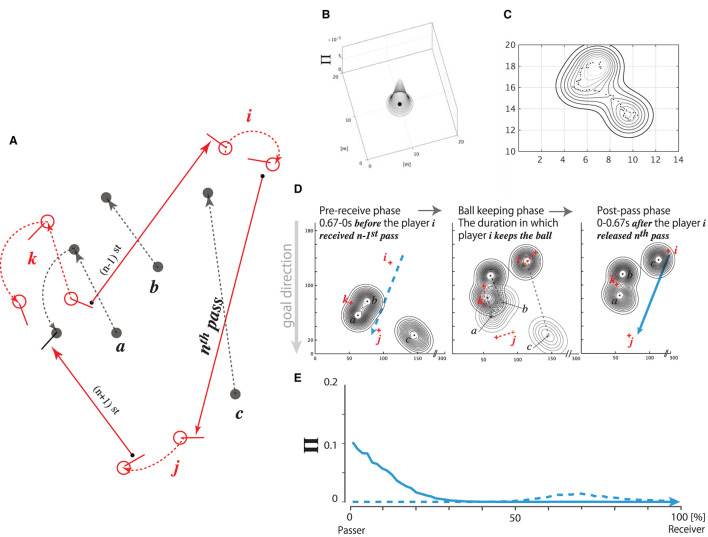
Concept of defensive pressure (Π) distribution. **(A)** Sequence from (*n*−1)st to (*n*+1)st passes fed from player *k* up to *i* and up to *j*. Attackers' positions and their movements (red open circles, each with a stick and red dotted arrows; denoted as *i*, *j*, and *k*), and defensive players' positions and their movements (black filled circles, each with a black dotted arrow; denoted as *a*, *b*, and *c*). Each trajectory, for the (*n*−1)st, *n*th, and (*n*+1)st passes, is represented by a red solid arrow ending with an arrowhead. **(B–E)** Procedure for calculating the fluctuation of defensive pressure on the pass trajectory. **(B)** 3D Π distribution pressed by a defensive player positioned at an arbitrary position (for example, [10,10] in the figure) on the pitch coordinate. **(C)** Pressure distribution pressed by a defender moving along the trajectory of dots in a given duration (*dt* seconds). **(D)** Three defenders' pressure distributions (player *a*, *b*, and *c*, represented by black dots with contour) and attackers (player *i*, *j*, and *k*, diagrammed by red crosses) in the three action phases of the attacker *i*: pre-receive phase, ball-keeping phase, and post-pass phase. *dt* = 0.67 s for both the pre-receive and post-pass phases. **Left**: Attacker *i* is to receive the (*n*−1)st pass released by attacker *k*, and the three defenders are pressing on the pitch during the pre-receive phase. Blue broken arrow represents the *n*th pass released by attacker *i* up to attacker *j* in the *postpass* phase. **Center**: Attacker *i* is keeping the ball, and the defenders/attackers are moving to intercept/thread the *n*th pass, respectively. The pressure distributions are moving from a sparse contour plot to a dense plot during this phase. **Right**: Attacker *i* releases the *n*th pass to attacker *j* being pressed by the defenders in the post-pass phase (*dt* = 0.67 s). Blue solid arrow represents the trajectory of the *n*th pass. Note that the *n*th pass trajectory is pressed differently in the pre-receive and post-pass phases. **(E)**: Π distribution on the *n*th pass trajectory caused by the positions of the defenders in the pre-receive (blue broken plot) and post-pass (blue solid plot) phases. The values “0” and “100%” in the abscissa denote the positions of passer *i* and receiver *j*, respectively.

Figure 2A depicts a chain of (*n*−1)st to (*n*+1)st passes successfully thread from players *k*, *i*, and *j*. It can be hypothesized that player *i* decided upon the *n*th pass trajectory depending on the defensive pressure on the pitch distribution during the phase in which player *i* received an (*n*−1)st pass fed by player *k*. Such a prediction would be necessary for the decision of all passing behaviors, except for those released from out of bounds. Based on this hypothesis, we calculated the defenders' pressure distribution (Kijima et al., 2014) on the forward pass trajectory for two phases, i.e., *before* and *after* the pass release.

In the first step of the calculation, we determined each defensive player's 2D position on the pitch. The pressure distribution was calculated based on the hypothesis that the player intensively pressed 1 m around himself on the pitch, unlike on the area beyond this range (for a typical example, see Figure 2B, and for a detailed calculation, see Equation (1) in the Additional Requirements).

Suppose that each of the three defenders moved in each direction on the pitch for a certain duration *dt*, as diagrammed in Figure 2C and formulated in Equations (2) and (3) in the Additional Requirements. In addition, suppose that the defenders were pressing on the *n*th pass trajectory within *dt*. We hypothesized that *dt* =0.67 s, which was the minimum time at which the ball traveled from the stick surface of the passer to that of the receiver, as measured in the experiment. Therefore, we postulated that the two phases, i.e., the pre-receive and post-pass phases, are critical to the pass-trajectory decision of the passer. Herein, the pre-receive phase corresponded to the phase 0.67 s before the passer received the previously released pass, whereas the post-pass phase corresponded to the 0.67-s duration after the moment at which the passer released the ball.

The three contour plots in Figure 2D illustrate typical examples of the defensive pressure distribution on the trajectory of the *n*th pass depicted in Figure 2A. The left panel represents the defensive pressure distribution during the pre-receive phase, which spanned the 0.67-s duration before the moment at which player *i* received the (*n*−1)st pass released by player *k*. Immediately after player *i* received the ball from player *k*, player *i* kept the ball for a given time, i.e., the ball-keeping duration, as depicted in the center panel. Meanwhile, the post-pass phase spanned the 0.67-s duration after the moment at which player *i* released the *n*th pass to player *j*, as depicted in the right panel. Each long vertical dotted line on the left panel and the solid arrow on the right panel show the same trajectory as that of the *n*th pass released by player *i* to player *j*. For the pre-receive and post-pass phases, we separately calculated the defensive pressure distributions and their effects on the trajectory of the *n*th pass.

The data indicated by each of the broken and solid lines in Figure 2E represent an example of the defensive pressure distributions Π projected onto the *n*th pass trajectory depicted in Figure 2A in the pre-receive and post-pass phases, respectively. Note that each corresponds to a broken blue arrow in the left panel of Figure 2D and a solid blue arrow in the right panel of Figure 2D. A high Π value in the 0% pass-trajectory region (near the release point of the pass) indicates that the defenders pressed on the position near the passer, whereas such a value on the 100% pass-trajectory region (the endpoint of the pass) indicates that the defenders pressed on the receiver's position. A slightly high Π value in the broken plot around the 70% pass-trajectory region, as shown in Figure 2E, indicates that the defenders pressed on a position 70% away from player*i* (passer) on the pass trajectory in the pre-receive phase, whereas an extremely high value in the solid plot in the 0–10% region indicates that the defenders moved to intensively press onto the passer in the post-pass phase.

To reveal group differences in the spatial–temporal parameters of the collective action of the players that causes not only pass chaining but also breaking, it is insufficient to analyze the effects only on all passes including successful and failed passes together. Therefore, the analysis was conducted in the following step-by-step manner. First, the defensive pressure distribution on both the successful- and failed-pass trajectories were pooled into a single dataset, and a 3-way mixed design ANOVA involving the groups (2: Ex, Im), action phases of the passer (2: pre-receive, post-pass), and pass-trajectory regions (10: 0–10, 10–20, … 90–100% points of pass trajectory) was conducted with repeated measurements for the latter two parameters. Second, the data were divided into two datasets for the successful-pass trajectory and failed-pass trajectory, and a mixed-design 3-way ANOVA involving the groups (2: Ex, Im), action phases of the passer (2: pre-receive, post-pass), and pass-trajectory regions (10: 0–10, 10–20, … 90–100% points of pass trajectory) was conducted on each dataset, i.e., for the successful and failed passes. When any violations to sphericity were found, we collected *p*-values using the Greenhouse-Geisser procedure. The Bonferroni method was used for multiple comparison. As assumed in the analysis of ball-keeping duration, when the size of the effect $\eta_{p}^{2}$ was equal to or larger than 0.14 and 0.06, we assumed that it indicated a sufficiently large and medium effect, respectively.

## Results

### In-play Time and Technical Action

Table 2 shows the between-group differences in-play time, in the numbers of successful and failed passes, in the number of shots, and in the number of turnovers per game. The result of the Mann–Whitney U test indicated that there were no significant differences in the in-play time and in the number of the turnovers, whereas there were significant differences in both the numbers of successful and failed passes (successful: *z* = 3.68, *p* < 0.01, *r* = 0.75; failed: *z* = 2.32, *p* = 0.02, *r* = 0.47). The Ex group more frequently succeeded and less frequently failed to thread a pass. Furthermore, the number of shots of the Ex group was significantly larger (*z* = 2.28, *p* = 0.02, *r* = 0.46).

Thus, the players in the Ex group succeeded in threading a pass more frequently during the same in-play time than those in the Im group. Therefore, in the Ex group, the attack–defense deadlock (or equilibrium) state fluctuated slightly and at a higher frequency compared to that in the Im group. Furthermore, in the Ex group, the attack–defense deadlock state tended to break more frequently into an advantageous state to the attacking side because the Ex players shot more frequently compared to the Im group, although the turnover frequency was equal between the groups.

### Ball-Keeping Duration

**Figure 3:**
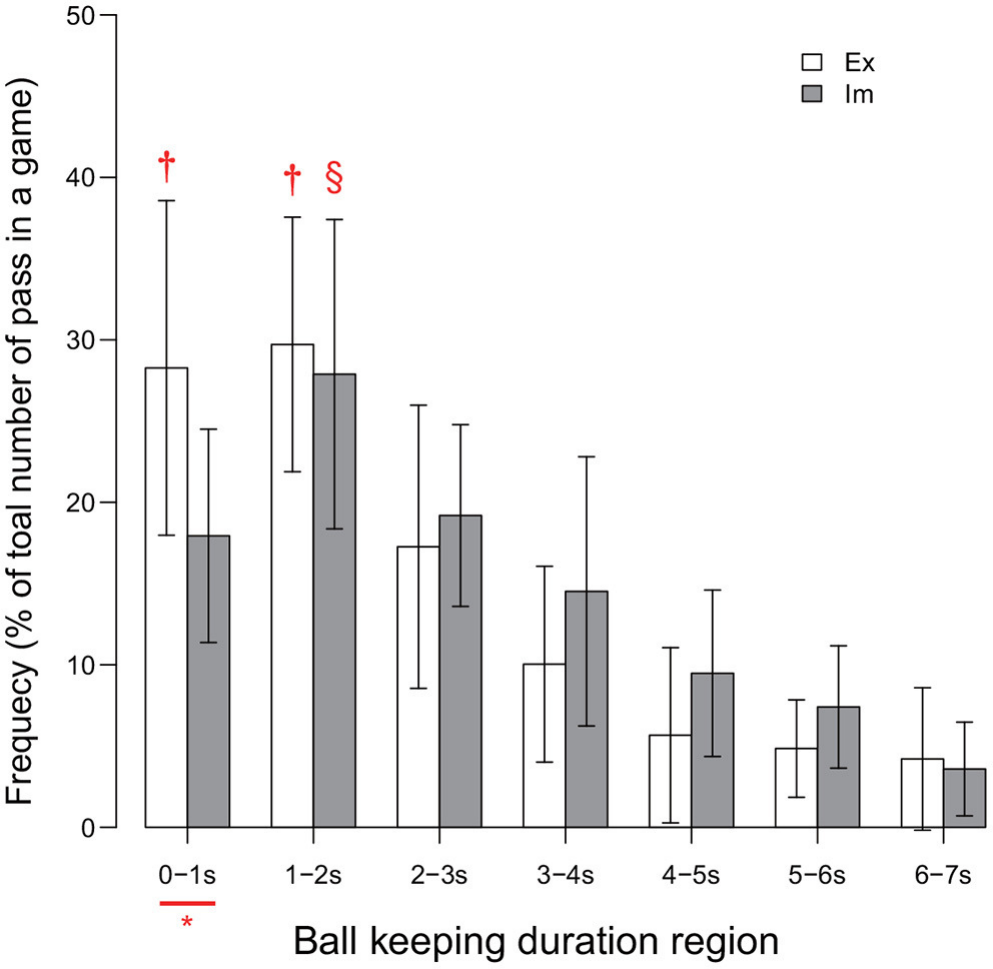
Distribution of ball-keeping time. * indicates significant difference between the groups. Each of the two † in each of the two regions for the Ex group indicates a significantly higher frequency than those in all other regions for the Ex group. Symbol § for the Im group indicates that the data were significantly higher than those of all other region. Level of statistical significance was set to p < .05.

The between-group difference in the ball-keeping duration is shown in Figure 3. The result of the ANOVA revealed the significant effect of the interaction between the group and the ball-keeping duration region [*F*_(3.75, 82.60)_ = 2.76, *p* = 0.04, $\eta_{p}^{2}=0.11$]. Analysis of this interaction revealed that the frequency of ball releases within durations less than 1.0 s was significantly higher for the Ex group than for the Im group [*F*_(1, 22)_ = 7.88, *p* = 0.01, $\eta_{p}^{2}=0.26$]. Moreover, the simple main effect of the ball-keeping duration region was significant for both the Ex [*F*_(3.26, 35.89)_ = 23.69, *p* < 0.01, $\eta_{p}^{2}=0.68$] and Im groups [*F*_(3.14, 34.52)_ = 15.87, *p* < 0.01, $\eta_{p}^{2}=0.59$]. For the Ex group, the frequency of ball releases in less than 1 s was equal to the frequency of ball releases in durations within the range 1–2 s. On the other hand, for the Im group, the frequency of the passer releasing the ball in less than 1 s was less than the frequency of the passer releasing the ball in durations within the range 1–2 s. In addition, the players in both groups released the ball in less than 2 s (i.e., sum of the frequencies for the 0–1 and 1–2 s regions) in half of all cases in which the players kept the ball (i.e., ≈57% for the Ex group and ≈45% for the Im group).

This series of results indicates that the players in the Ex group released the ball within a relatively shorter duration compared to that for the Im group. This trend will lead to a decrease in the risk of turnover and will also stabilize the chaining of the pass, as shown by the significant between-group differences in the numbers of successful and failed passes, as illustrated in Table 2.

### Defensive Pressure Distribution on Pass Trajectory

#### Structure of Collective Pass-Chaining Action Common to Ex and Im Group

If the ball-keeping duration is extended, the cognitive process of the player to decide the next pass trajectory will tend not to be influenced by the defenders' action reacting to the preceding pass. Therefore, in this analysis, we investigated the effect of the distribution of defensive pressure (abbreviated as Π, as shown in Figure 2E) on the forward pass trajectory only after a release following a ball-keeping duration less than or equal to 2 s. The percentage values of forward passes included in the following analysis was 57% (Ex group) and 73% (Im group) of the total number of times when the respective players kept the ball for less than 2 s.

**Figure 4:**
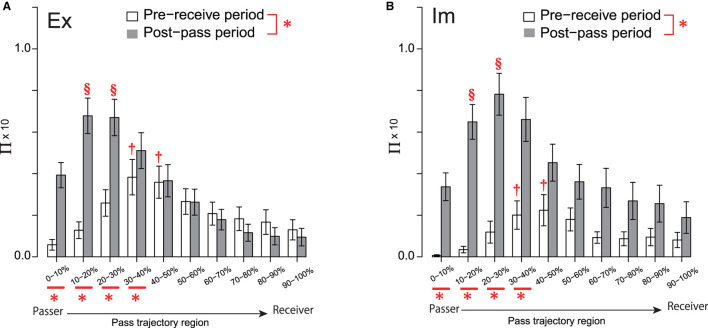
Π distribution on the pass trajectory for the Ex **(A)** and Im **(B)** groups. Significant differences between the two phases and those among the pass-trajectory regions were equivalent for the two groups, because the group had no significant (simple) main effect. Each of the two † in each of the two regions for both groups indicates a significantly higher frequency than those in all other regions, except for the 20%–30% and 50–60% regions. Each of the two § in each of the two regions for both groups indicates significantly higher values than in those of all other regions, except for the 30–40% region. *under the abscissa indicate a significant simple main effect of the group. *bonding two elements of the legends indicate a significant simple main effect of the phase. Level of statistical significance was set to p < .05.

In the first step of the analysis, the 3-way ANOVA involved the groups (2: Ex, Im), action phases of the passer (2: pre-receive, post-pass), and ball trajectory regions (10: 0–10, … 90–100% of the pass trajectory) and was conducted on the dataset shown in Figures 4A,B. The interaction between the group and the action phase of the passer was significant [*F*_(1, 74)_ = 5.52, *p* = 0.02, $\eta_{p}^{2}=0.07$]. Whereas the simple main effect of the group for both the pre-receive and post-pass phases was not significant, the simple main effect of the action phase of the passer was significant for both the Ex [*F*_(1, 43)_ = 7.88, *p* = 0.01, $\eta_{p}^{2}=0.15$] and Im [*F*_(1, 31)_ = 17.48, *p* < 0.01, $\eta_{p}^{2}=0.36$] groups. As shown in Figures 4A,B, the effect of Π on the overall region in the post-pass phase was more intensive than that in the pre-receive phase for both the Ex and Im groups.

In addition, the effect of the interaction between the action phase of the passer and the pass-trajectory regions was also significant [*F*_(2.88, 212.77)_ = 16.68, *p* < 0.01, $\eta_{p}^{2}=0.18$]. Analysis of the interaction revealed that the simple main effect of Π in each of the four pass-trajectory regions spanning 0–10 to 30–40% was significant. In each of these four regions, Π in the post-pass phase was higher than that in the pre-receive phase. The simple main effect of the pass-trajectory region was also significant in both the pre-receive [*F*_(2.39, 176.71)_ = 8.18, *p* < 0.01, $\eta_{p}^{2}=0.10$] and post-pass [*F*_(2.92, 216.21)_ = 27.69, *p* < 0.01, $\eta_{p}^{2}=0.27$] phases. *Post-hoc* analysis showed that the Π values in the two regions spanning 30% to 50% were more intensive than those in 0–10%, 10–20%, and the four regions spanning from 70 to 100% in the pre-receive phase. On the other hand, in the post-pass phase, the Π values in two regions, i.e., 20–30 and 30–40%, were significantly higher than those in 0–10% and in the six regions spanning 40 to 100%.

This series of results indicates that the Π distribution on the overall pass trajectory was evidently higher in the post-pass phase, especially in the regions around the passer's position; however, defenders systematically press on the midpoint of the pass trajectory even in the pre-receive phase. This systematic trend of the pressure distribution pattern that is common to both groups suggests that it is common for both groups to continually chain the pass, resulting in fewer fluctuations in the attacker–defender deadlock states.

#### Group Difference in Breaking Process of Pass Chaining

**Figure 5:**
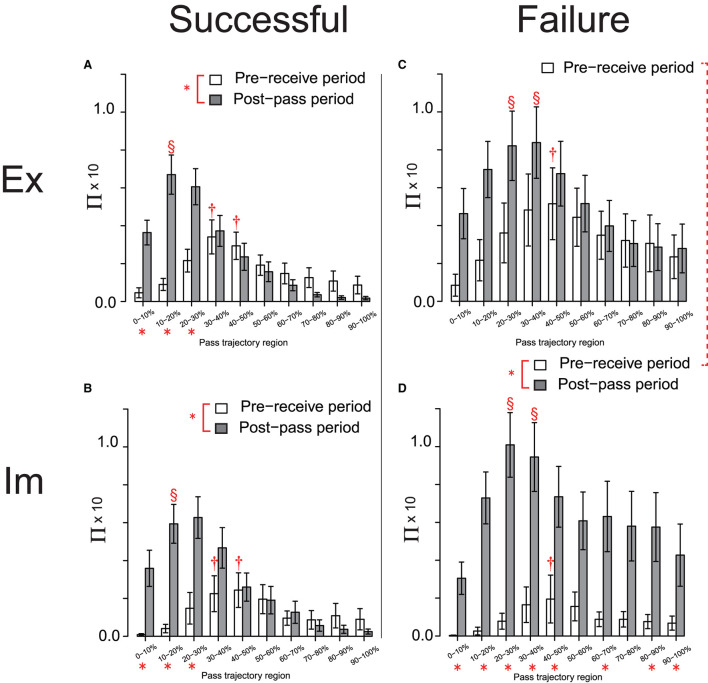
Π distribution on the pass-trajectory regions calculated for the Ex group for successful **(A)** and failed **(C)** passes, and those for the Im group [**(B, D)**, respectively]. *indicate significant simple main effects of the action phase of the attackers. The red broken line connecting the legends indicates the trend for the significant simple main effects of the group (*p* = 0.09). Results of the *post-hoc* test on the simple main effect of the pass-trajectory regions in each action phase are represented by † and §. In **(A)** and **(B)**, † indicates significantly higher values than those in 0–20%, and § indicates a significantly higher value than those in all regions except for 10–20%. In **(D)**, † indicates significantly higher values than those in 0–20% and the two § indicate significantly higher values than those in all regions except for 10–20% and 40–60%.

In the next step of the analysis, each dataset of the Π distribution for the Ex and Im groups, shown in Figures 4A,B, respectively, were divided into two sets based on the effects on the trajectories of the successful and failed passes, as shown in Figures 5A–D, respectively. A 3-way ANOVA involving the groups, action phases of the passer, and pass-trajectory regions was conducted on the datasets for successful passes in both groups, as shown in Figures 5A,B. The results revealed no significant between-group differences, although the main effect of the action phase of the passer [*F*_(1, 48)_ = 13.93, *p* < 0.01, $\eta_{p}^{2}=0.22$] and that of the pass trajectory region [*F*_(2.65, 127.22)_ = 19.61, *p* < 0.01, $\eta_{p}^{2}=0.29$] was significant; in addition, the interaction between the action phase of the passer and the pass-trajectory region was significant [*F*_(2.70, 129.66)_ = 14.46, *p* < 0.01, $\eta_{p}^{2}=0.23$]. The simple main effect of the action phase of the passer and that of the pass-trajectory region are shown in Figures 5A,B.

However, the result of 3-way ANOVA conducted using the Π distribution on the failed-pass trajectory (Figures 5C,D) revealed that the effect of the interaction of the group and the action phase was almost significant [*F*_(1, 24)_ = 3.73, *p* = 0.07, $\eta_{p}^{2}=0.13$]. As determined *via* the analysis of this interaction, the effects of Π distribution on the overall pass trajectory in the pre-receive phase tended to be more intensive in the Ex group compared to in the Im group [*F*_(1, 24)_ = 3.03, *p* = 0.09, $\eta_{p}^{2}=0.11$], as shown by the dotted connection line between the legends in Figures 5C,D. In addition, the simple main effect of the action phase of the passer was significant only for the Im group [*F*_(1, 12)_ = 14.17, *p* < 0.01, $\eta_{p}^{2}=0.54$], which indicates that the effect of the Π distribution on the overall regions of the pass trajectory in the post-pass phase was significantly more intensive than that in the pre-receive phase [*F*_(1, 12)_ = 14.17, *p* < 0.01, $\eta_{p}^{2}=0.54$]. In addition, the interaction between the action phase of the passer and the pass-trajectory region was significant [*F*_(2.62, 62.89)_ = 4.08, *p* = 0.01, $\eta_{p}^{2}=0.15$]. The simple main effect of the action phase of the passer and that of the pass-trajectory region in each of the Ex and Im groups are shown in Figures 5C,D, respectively.

Meanwhile, there was no between-group differences in the Π distribution on the successful passes. This result indicates that the temporal (i.e., of the action phase of the passer) and spatial (i.e., of the pass trajectory region) effects of the defensive pressure were equivalent between the groups and also equivalent to that for the combined data shown in Figure 4. Thus, the basic structures of the defensive pressure distributions that emerged in pass chaining were common for both groups. Herein, the defenders predictively pressed on the mid-point of the trajectory of the forthcoming pass when the passer received the previous pass and then moved to press on the passer himself when the passer released the ball to the next player. However, specifically only for failed passes, both the Ex and Im defenders tended to press more intensively on the overall pass trajectory compared to when their actions later resulted in successful passes, especially as the passer released the ball. Moreover, and especially for the Im group, extremely intensive defensive pressure affected the overall pass trajectory, especially as the passer released the ball.

Thus, the results reveal between-group differences in the cause of the breakage of the pass-chaining actions performed by the attackers. For the Ex players, this cause was primarily a holistic increase in the defensive pressure over the entire duration of the passing action, including the pre-receive and post-pass phases, relative to that on the trajectory of a successful pass. On the other hand, for the Im players, this cause was primarily the intensive increase in defensive pressure skewed when the passer released the ball to the next player. In addition, the magnitude of Π on the failed-pass trajectory near the position at which the next player received the pass was more intensive compared to that on the corresponding successful-pass trajectory (i.e., regions spanning approximately 50–100%; see Figures 5C,D).

### Ball-Keeping Durations in Successful and Failed Passes

**Figure 6:**
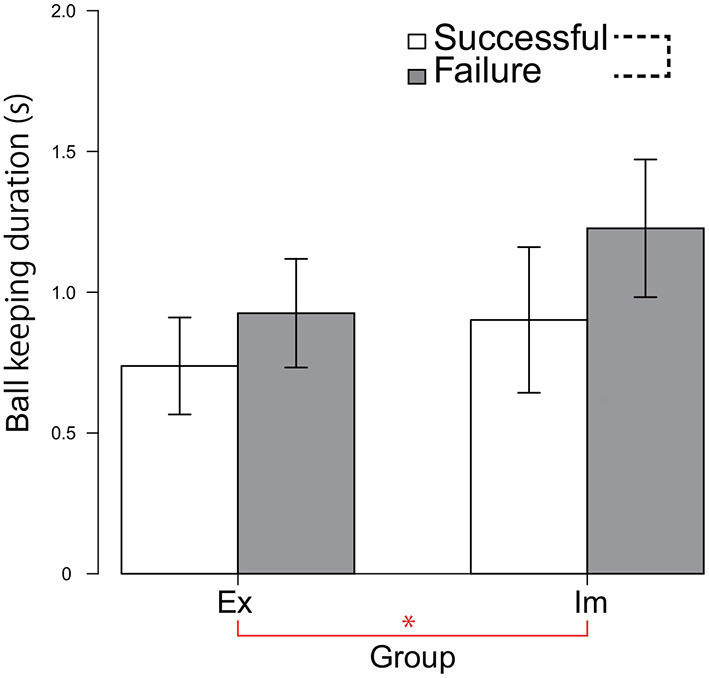
Ball-keeping duration for each of the successful and failed passes. The value indicates the mean of the median calculated for each of the six game data. The data for one game in both the Ex and Im groups was excluded owing to missing data. * indicates a significant main effect of the group. The black dotted line connecting two elements of the legend indicates the trend (*p* = 0.09) for the significant main effect of the pass consequence.

Particularly when the Im players failed to pass, the pressure distribution increased intensively at the moment at which the passer released the ball. Furthermore, although the trend was not statistically significant, the Π distribution for failed forward passes in the pre-receive phase was less intensive in the Im group compared to the Ex group. We hypothesized that such divergence of Π distribution between the pre-receive and the post-pass phase observed only for the Im group data was due to the extensions in the ball-keeping duration. Therefore, we tested this hypothesis by performing a 2-way ANOVA involving the groups and the pass consequences on the ball-keeping duration dataset, which was restricted to values less than 2 s (i.e., limited to the duration of the ball-keeping phase, which follows/is followed by the pre-receive/post-pass action included in the Π distribution analysis shown in Figures 4, 5). This hypothesis was partially supported by the results shown in Figure 6. The main effect of the group was significant, indicating that the ball-keeping duration was longer for the Im group [*F*_(1, 8)_ = 8.60, *p* = 0.02, $\eta_{p}^{2}=0.52$]. Furthermore, there was a tendency for these values to be longer when the passer failed to thread the pass, although the main effect of the action phase of the passer was slightly less than what is considered statistically significant [*F*_(1, 8)_ = 3.67, *p* = 0.09, $\eta_{p}^{2}=0.31$].

## Discussion

The importance of this present research was to reveal the temporal and spatial orderly pattern of the lower-order pass-chaining states using mathematical constructs of the defensive pressure distribution. Compared to those belonging to the Im group, players in the Ex group tended to end the pass chain by shooting the ball, and their probability of being successful in threading the pass tended to be higher, as shown in Table 1. In addition, the probability of failure in threading the pass was lower for the Ex group. Therefore, the attack/defense equilibrium state in the Ex group fluctuated less than that in the Im group, concurrently, as shown by the relative successful pass frequency, and superior shooting opportunities among the Ex group players.

The players recruited for this research are regarded as advanced collegiate-level hockey players at the Japanese national competitive level. These advanced collegiate players, including not only the Ex but also the Im players, are able to release their passes, which determine where the defensive pressure is less intensive. Because the pitch used in the experiment was sufficiently cramped to enable the defenders to easily press the passers, defenders were already able to press on the midpoint of the pass trajectory by 0.67 s before the passer (e.g., attacker *i* in Figure 2A), received the pass previously released by another attacker (e.g., attacker *k*). Moreover, in approximately half of all cases investigated in this study (as shown in Figure 3), the passer released the ball for the next attacker in less than 2.0 s. Despite such a short ball-keeping duration, as shown in Figures 4A,B, the defenders quickly approached the passer to press intensively on the points at which the ball was released, by continuously and also systematically pressing on the pass trajectories of the forthcoming pass before the passer received the ball. Therefore, the defenders were able to quickly press on the passer when the passer moved the ball around his foot or released the pass. On the other hand, with regard to the attacking players, the passer released the ball before the moment at which he had to collapse with the defenders and also at the moment the receiver was free from the defenders' pressure, as illustrated in Figures 4A,B. Furthermore, the same results obtained in the analysis of the successful passes for both the Ex and Im groups (Figures 5A,B) suggest that all players in both groups were able to maintain such a state of pass chaining.

On the other hand, for both the Ex and Im groups, when the pass-chaining process was broken, the ball-keeping duration was increased, as shown in Figure 6. However, there was an essential difference between the groups in terms of the temporal and spatial order in breaking the pass chain. In the Ex group, the state was broken when the defensive pressure on the pass course was increased within the overall duration encompassing both the pre-receive and post-pass phases; that is, the time from 0.67 s *before* the passer received the previous pass, to 0.67 s *after* the moment at which the ball was released from the passer's stick surface. By contrast, in the Im group, the chain was broken when the defensive pressure increased greatly only in the 0.67 s *after* the ball was released. Ball control skill is one of the necessary abilities that determine a player's game performance (Elferink-Gemser et al., 2004). However, as shown in Table 1, it is difficult to hypothesize that differences in the pass accuracy or in the quickness to receive/release the ball determine this between-group difference. Instead, it can be postulated that the state was determined by the spatial–temporal relationship between the alignment of the defensive players and the attacker's state, as revealed in research studies on rugby (Passos et al., 2008; Correia et al., 2012) or soccer (Clemente et al., 2013).

In the Ex group, the pass chain was broken when the defense put more intensive pressure during the entire duration, including the pre-receive and post-pass phases. This increase in the defensive pressure emerged because both the attackers and defenders were reciprocally attracted toward each other. Although further analysis is beyond the scope of the present research, this hypothesis can be tested based on a time series of the sizes of the areas dominated by the attack and defense sides (Yokoyama and Yamamoto, 2009). On the other hand, in the Im group, the chain was broken when the defense put extremely intensive pressure on the passer at the moment that the ball was released. When the players failed to thread the pass, the means of the ball-keeping durations were 1.02 s for the Ex group and 1.35 s for the Im group, as shown in Figure 6. Therefore, when their pass chains were broken by the defenders, the Im passers had kept the ball around their foot 0.33 s longer than the Ex passers did. If this delay had led to the pass-chain breakage in the Im group, then the ball-keeping time should be suppressed to less than 1 s to ensure that the chain of the pass will be continued. When the passers in the Ex group succeeded to thread their passes, their ball keeping duration was 0.81 s and that for the Im group was 0.99 s. Therefore, it can be inferred that a time constant of 1 s more or less for the pass exchange/interception will constrain the dynamical order stabilization of the hockey game in national top-level competitions.

Thus, we inferred the temporal and spatial constraints that emerge for pass chaining and game momentum, and determined that the processes of breaking this state were different between the Ex and Im groups by capturing the temporal constraints of the order of the collective behavior of the hockey players. One can hypothesize that, basically, the temporal and spatial constants confirmed in the current experiment would equally afford Ex and Im players' actions since temporal and spatial defensive pressure patterns on the succeeded pass is equivalent in the Ex and Im groups. Then, what was the cause of the delay in Im players' receive–pass action? We speculate that there may have been a lack of *shared affordance*, which would have emerged in the mutual perception of the players attuning to affordance *of* others and affordance *for* others (Fajen et al., 2008; Araújo et al., 2016) during unexpected change of the defensive pressure distribution. Theoretically, it can be hypothesized that this would enhance the assembly of the synergy (Riley et al., 2011) and, consequently, degenerate the system of collective action of the players (Davids et al., 2006).

Practically, it has been confirmed that such mutual adjustment can be learned by the college student *via* through ten-weeks training (Sampaio and Maçãs, 2012). Based on the dynamical systems theory, it can be supposed that adjusting the pitch size or number of players would contribute to enhancing the assembly of the synergy of the Im players (Chow et al., 2011, 2015; Renshaw et al., 2019); however, such a procedure, wherein only the density of the players is adjusted, may be insufficient to improve the game performance of already excellent players (Owen et al., 2004). If we are to propose a small-sided game environment to enhance the pass-chaining performance of the Im attackers based on our findings, it would be that the coach should increase the density of the players sufficiently to enable the defenders to press on the pass trajectory in a temporal and spatial order equivalent to that shown in Figures 4, 5A,C. Furthermore, for the Im attackers to learn to adapt to this pressure, the coach should rush to the passer when he/she receives the previous pass, and press intensively to the passers sufficiently to force the passer to release the ball in 1 s. It would be important for the coach to know and, moreover, to be able to repeat the timing or the distance to rush to the passer, in order to reproduce “unexpected events” for Im players that might actually occur in top-level competitive situations in which Ex players usually play. For the Im attackers to adapt to this perturbation, they must be able to reproduce the dynamical equilibrium state of the attack–defense deadlock that emerged in the Ex group's games. As a consequence, both the attacking and defensive abilities will improve in such a state involving actively fluctuating collective behaviors among the hockey players.

## Data Availability Statement

The raw data supporting the conclusions of this article will be made available by the authors, without undue reservation.

## Ethics Statement

The studies involving human participants were reviewed and approved by Research Ethics Committee of the Faculty of Education, University of Yamanashi. Written informed consent to participate in this study was provided by the participants' legal guardian/next of kin.

## Author Contributions

TM and AK conceived the experiment and conducted the experiment. TM, MO, and AK analyzed the results. All authors reviewed the manuscript. All authors contributed to the article and approved the submitted version.

## Funding

This work was supported by JSPS KAKENHI (Grant Numbers 20H04090 and 20K21885).

## Conflict of Interest

The authors declare that the research was conducted in the absence of any commercial or financial relationships that could be construed as a potential conflict of interest.

## Publisher's Note

All claims expressed in this article are solely those of the authors and do not necessarily represent those of their affiliated organizations, or those of the publisher, the editors and the reviewers. Any product that may be evaluated in this article, or claim that may be made by its manufacturer, is not guaranteed or endorsed by the publisher.

## Additional Requirements

**Procedure for calculating defensive pressure distributions**. The intensity with which the *i*th player at position (*x*_*i*_, *y*_*i*_) influences the given pitch position (*x, y*) is indicated by the function *p*_*i*_(*x, y*) defined by

(1)
$$\begin{array}{r} {\text{p}}_{\text{i}}\left(\text{x},\text{y}\right)=\frac{1}{2\text{πσ}}{\text{e}}^{\frac{\text{x}-{\text{x}}_{\text{i}}}{\text{σ}}+\frac{\text{y}-{\text{y}}_{\text{i}}}{\text{σ}}}, \end{array}$$

where σ denotes the critical distance with which an individual player is able to defend effectively. The sum of *p*_*i*_ over three defenders is designated as follows:

(2)
$$\begin{array}{r} {\text{P}}_{\text{t}}\left(\text{x},\text{y}\right)=\overset{\text{N}}{\underset{\text{i}=1}{\sum }}{\text{p}}_{\text{i}}\left(\text{x},\text{y}\right)\text{ withN}=3, \end{array}$$

Which determines the degree of domination of the three defenders at position (*x, y*) at moment *t*. The contour plot shown in Figure 2B graphically demonstrates the defensive pressure distribution defined by the position of a defender ([10,10]) and a parameter σ. It is reasonable to set σ = 1 m, because in an actual game, the players intercept the ball using a stick approximately 1.05 m long. The total of the three defenders' pressures at position (*x, y*) from the moment of the *t*-th data frame to the *t*+Δ*t*-th frame is defined by

(3)
$$\begin{array}{r} \text{Π}\left(\text{x},\text{y}\right)=\overset{\text{t}+\text{Δt}}{\underset{\text{j}=\text{t}}{\sum }}{\text{P}}_{\text{j}}\left(\text{x},\text{y}\right). \end{array}$$

The time constant Δ*t* was set to be 20 video frames (≈0.67 s, because the data were measured at 30 Hz), which was approximately equal to the shortest duration of travel of the ball from a passer to a receiver among our data. Typical examples of the contour plot of Π calculated for the pre-receive- and post-pass phases are shown in Figures 2D,E.
